# Sleep and Psychosocial Characteristics of Children with Narcolepsy According to Their Intellectual Profile: A Case–Control Study

**DOI:** 10.3390/jcm11164681

**Published:** 2022-08-10

**Authors:** Marine Thieux, Min Zhang, Agathe Marcastel, Alice Poitrinal, Fanny Vassias, Aurore Guyon, Olivier Revol, Stephanie Mazza, Anne Guignard-Perret, Patricia Franco

**Affiliations:** 1 Lyon Neuroscience Research Center, INSERM, U1028, CNRS, UMR5292, 69500 Lyon, France; 2 Pediatric Sleep Unit, Department of Pediatric Clinical Epileptology, Sleep Disorders and Functional Neurology, Hôpital Femme Mère Enfant, Hospices Civils de Lyon, 69500 Lyon, France; 3 Department of Developmental Psychology, Hôpital Femme Mère Enfant, Hospices Civils de Lyon, 69500 Lyon, France; 4 Research on Healthcare Performance RESHAPE, INSERM U1290, Université Claude Bernard Lyon 1, 69500 Lyon, France

**Keywords:** cognition, sleepiness, polysomnography, intellectual quotient

## Abstract

Adequate intellectual abilities are a protective factor for psychosocial adjustments in chronic disorders. The main objective of this study was to assess the cognitive abilities, sleep, and psychosocial characteristics of children with narcolepsy compared to controls, according to their intellectual profile. Children underwent a polysomnography, completed an intellectual ability assessment, and filled out standardized questionnaires. The group with an intelligence quotient (IQ) in the area of high intellectual potential (high IQ, HIQ) consisted of 25 children with narcolepsy (HIQ-N, 40% boys, median age 11.5 years, 48% with obesity, 60% under treatment) and 25 controls (HIQ-C, 68% boys, median age 11.7 years). Compared to HIQ-C, HIQ-N had a lower perceptual reasoning index and fewer conduct disorders. The group with an IQ in the normal range (NIQ) consisted of 22 children with narcolepsy (NIQ-N, 55% boys, median age 12.1 years, 59% with obesity, 64% under treatment) and 21 controls (NIQ-C, 68% boys, median age 10 years). NIQ-N presented the same intellectual profile as NIQ-C but reported more school difficulties. In children with HIQ, those with narcolepsy appear to have a different cognitive profile than controls. NIQ seems to predict a greater impact of narcolepsy on daily-life functioning.

## 1. Introduction

Narcolepsy is a rare chronic sleep disorder characterized by excessive diurnal sleepiness and irresistible sleep attacks associated with cataplexies, hypnagogic and hypnopompic hallucinations, sleep paralysis, and disturbed nocturnal sleep [1,2]. In more than half of the cases, narcolepsy begins before the age of 18 years [3]. The prevalence of obesity, night eating, sleep talking and drunkenness, parasomnia, and symptoms of attention deficit hyperactivity disorders at diagnosis is higher in children than in adults [4]. Due to this wide range of manifestations, children are at risk of being misdiagnosed [5], with a delay in diagnosis typically exceeding 10 years after symptom onset [6].

Since childhood is a critical period for neurobiological, cognitive, social, and psychological development, narcolepsy can have serious consequences in children, leading to a decrease in quality of life [7,8,9]. Cognitive abilities could play a major role in coping with narcolepsy and its repercussions, as it is the case in neurological, psychiatric, and chronic disorders [10]. Indeed, while the intelligence quotient (IQ) of children with narcolepsy is within the normal range [11,12,13], those with normal-to-low IQ have more psychiatric comorbidities [14], a feature also present in adults [15]. Our group has previously studied the characteristics of children with narcolepsy and those of controls according to their intellectual profile, i.e., high IQ (HIQ) versus normal IQ (NIQ) [16,17]. In children with narcolepsy, those with HIQ tended to have less school and behavioral difficulties than those with NIQ and Rapid Eye Movement (REM) sleep was positively correlated with IQ [16]. Furthermore, control children with HIQ had more REM sleep during the night than controls with NIQ [17]. To further decipher the impact of narcolepsy on sleep characteristics and its psychosocial repercussions while controlling for intellectual profile, children with narcolepsy were compared to controls using the same IQ range (i.e., HIQ and NIQ).

## 2. Materials and Methods

### 2.1. Population

Two groups of children were included in this retrospective study: 46 controls and 47 children with idiopathic narcolepsy [18,19,20] with or without cataplexy (Supplementary Materials, Figure S1).

#### 2.1.1. Control Children

Control children were recruited from both the ENSOM study (*n* = 26) and from the department of developmental psychology of the Lyon university hospitals, France (*n* = 20), where they consulted mainly for school orientation issues.

#### 2.1.2. Children with Narcolepsy

Children with narcolepsy were included at diagnosis, between 2010 and 2019, in the reference center for narcolepsy and rare hypersomnia of the department of pediatric clinical epileptology, sleep disorders and functional neurology of the Lyon university hospitals, France. The diagnostic procedure included sleep and wake monitoring as follows: a clinical examination with a pediatric sleep specialist, a polysomnography from 8 pm to 7 am followed by 4 or 5 standard Multiple Sleep Latency Tests (MSLT) at 9 am, 11 am, 1 pm, 3 pm, and 5 pm, which were ended after 20 min if no sleep occurred and after 15 min if sleep occurred [19]. Given that some patients were diagnosed before the validation of the international classification of sleep disorders (ICSD-3), the diagnosis of narcolepsy was consistent with the ICSD-2 criteria: complaints of excessive daytime sleepiness for at least 3 months, symptoms not better explained by other medical or psychiatric disorders, absence of secondary narcolepsy, presence of clear cut cataplexy and/or mean sleep latency during MSLT lower than 8 min and/or two or more sleep-onset REM periods [19]. Forty-two children with narcolepsy experienced cataplexies, 6 had sleep paralysis, and 16 experienced hallucinations. All the patients (except one with missing data) were HLA-DR-DQB1*06:02 positive, and hypocretin-1 was determined in 24 patients, in duplicate, from cerebral spinal fluid (CSF) samples [21]. They all had low CSF hypocretin-1 levels (i.e., <110 pg/mL) except one with intermediate levels (i.e., <200 pg/mL).

### 2.2. Procedure and Material

All children benefited from an interview with a pediatric sleep specialist (PF, AGP), a psychometric evaluation, and a one-night polysomnography (PSG). Age, sex, BMI (for children with narcolepsy) [22], socio-economic level of the parents, and school difficulties estimated by parents were collected.

#### 2.2.1. Psychometric Evaluation

**Figure 1:**
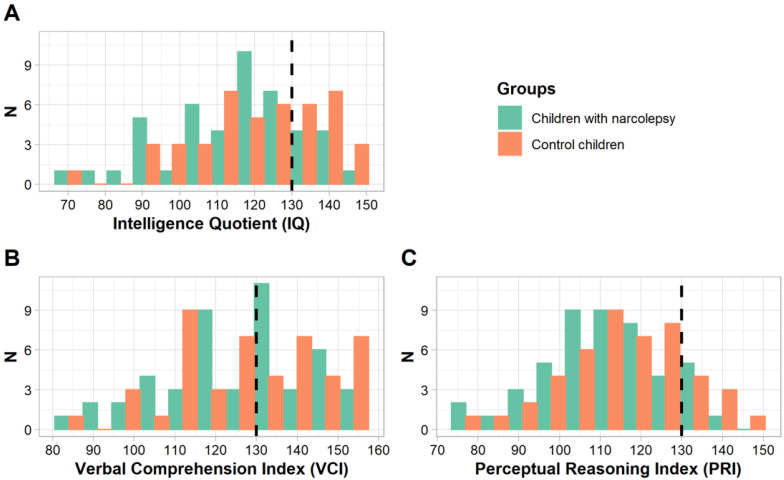
Histograms of the Weschler Intelligence Scale for Children (WISC) scores ((**A**) Intellectual Quotient; (**B**) Verbal Comprehension Index; (**C**) Perceptual Reasoning Index) in children with narcolepsy (green) and in control children (orange). The dashed lines represent the threshold scores for the area of high intellectual potential.

The psychometric evaluations were performed by experienced neuropsychologists using the Wechsler Intelligence Scale for Children (WISC) [23] providing the IQ which is the combination of the Verbal Comprehension Index (VCI), Perceptual Reasoning Index (PRI), Working Memory Index (WMI), and Processing Speed Index (PSI). Each score is normalized within each age group (mean 100, standard deviation 15). An absolute difference ≥15 between VCI and PRI is known as Significant Verbal Performance Discrepancy (SVPD). When the IQ is in the area of high intellectual potential (i.e., with a VCI, PRI, or IQ ≥ 130, representing more than 2 standard deviations from the mean of the normal distribution) [24,25] children were included in the HIQ group, otherwise children were included in the NIQ group (Figure 1).

#### 2.2.2. Questionnaires

Four questionnaires were used: the Adapted Epworth Sleepiness Scale (AESS) to assess sleepiness [26], the Insomnia Severity Index (ISI) for insomnia [27], the Child Depression Inventory (CDI) to assess depressive symptomatology [28], and the Revised Conners Parents Rating Scale (Conners) for behavioral and attention disorders [29] (Supplementary Materials). Apart from CDI, which was completed by children under parental supervision, all questionnaires were filled out by the parents. Questionnaires were then analyzed by experienced neuropsychologists under the supervision of sleep certificated specialists (PF, AGP, AG).

#### 2.2.3. Polysomnography

The PSG of the first hospitalization night was used for children with narcolepsy. For control children, the PSG was set up in the hospital and they were sent home to sleep overnight. The PSG included 8 electrodes referenced to the mastoids according to the 10–20 system, 2 electro-oculograms, 1 electromyography on the levator menti surface and left and right anterior tibialis muscles, an oral thermistor, thoracic and abdominal belts, an electrocardiogram, a transcutaneous oximeter, and a nasal cannula. The oral thermistor and nasal cannula were not used in controls as these children did not present clinical signs of obstructive sleep apnea. The pediatric criteria from the American Academy of Sleep Medicine were used for the visual scoring of sleep [19]. Total sleep time (TST), sleep efficiency, sleep and rapid eye movement (REM) latency, duration and percentage of each stage (N1, N2, N3, REM), arousal index, and wake after sleep onset (WASO) were collected. Obstructive apnea hypopnea index (OAHI), minimal oxygen saturation, index of desaturation ≥ 3%, periodic limb movement sleep index (PLMSI), and PLMSI ≥ 5/h were collected for children with narcolepsy.

### 2.3. Statistical Analysis

Statistical analyses were conducted using R software version 3.6.3 (Vienna, Austria) [30]. Continuous measures were expressed as median and range. Dichotomous and polytomous measures were expressed as number and percentage. Comparisons between groups of HIQ children with narcolepsy (HIQ-N) and HIQ controls (HIQ-C) or between NIQ with narcolepsy (NIQ-N) and NIQ controls (NIQ-C) were performed using Wilcoxon (W) or *t*-tests (t) for continuous measures according to the results of the Shapiro–Wilk tests (i.e., for the evaluation of the normality of the distribution) and the evaluation of the variance equality. Fisher’s exact tests (F) were used for dichotomous measures and the Chi2 test (X^2^) for polytomous measures. Results are reported with their 95% confidence intervals (CI) and statistical significance value was set to a *p*-value below 0.05.

## 3. Results

### 3.1. Children with HIQ

Fifty children were categorized in the HIQ group: 25 HIQ in the narcolepsy group (HIQ-N, 40% boys, median age 11.5 years) and 25 HIQ in the control group (HIQ-C, 68% boys, median age 11.7 years). There was no significant difference between groups in terms of age, sex, and parental socio-economic levels.

**Figure 2:**
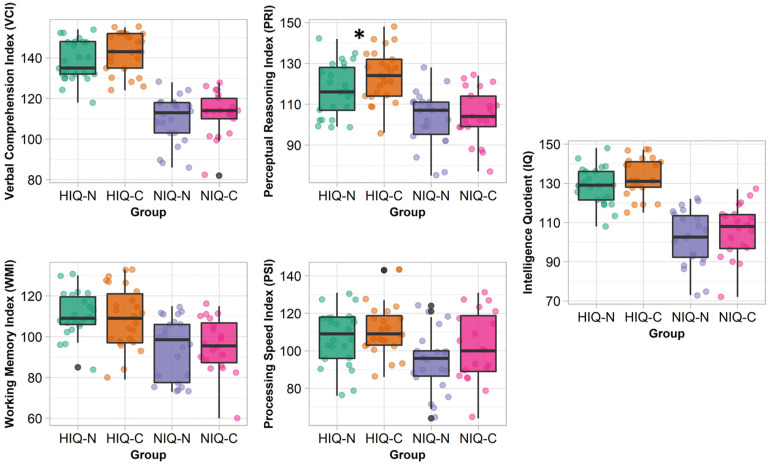
Verbal comprehension index (VCI), perceptual reasoning index (PRI), working memory index (WMI), processing speed index (PSI) and intelligence quotient (IQ) of the Weschler Intelligence Scale for Children (WISC) in each group (green: HIQ-N, orange: HIQ-C, purple: NIQ-N, and pink: NIQ-C). The central line of the boxplots represents the median of each score, the upper and lower parts represent the first and third quartiles. Each point represents the score of one child. Significant differences between groups of the same IQ range (HIQ-N vs. HIQ-C and NIQ-N vs. NIQ-C) are represented by a star: * *p* < 0.05.

Compared to HIQ-C, HIQ-N had a lower PRI (median 116 vs. 124, *p* = 0.047) (Figure 2), a higher AESS score (median 16 vs. 2, *p* < 0.001), more AESS pathological score (96% vs. 4%, *p* < 0.01), a lower Conners total score (median 15 vs. 33, *p* = 0.03), and a lower Conners subcomponent of conduct disorders score (median 43 vs. 51, *p* = 0.03).

HIQ-N had less TST (median 463 min vs. 522 min, *p* < 0.001), lower sleep efficiency (84% vs. 96%, *p* < 0.001), less stage 2 (median 178 min vs. 234 min, *p* < 0.001 and 39% vs. 45%, *p* = 0.04), more stage 1 (median 14% vs. 10%, *p* = 0.01), less stage 3 (median 90 min vs. 111 min, *p* = 0.02), shorter sleep (median 6 min vs. 28 min, *p* < 0.001) and REM (median 17 min vs. 131 min, *p* < 0.001) latencies, higher arousal index (median 12/h vs. 10/h, *p* = 0.04) and more WASO (median 92 min vs. 23 min, *p* < 0.001) than HIQ-C.

There was no other significant difference between groups. All characteristics are reported in Supplementary Material, Table S1.

### 3.2. Children with NIQ

Forty-three children were categorized in the NIQ group: 22 NIQ with narcolepsy (NIQ-N, 55% boys, median age 12.1 years) and 21 NIQ from the control group (NIQ-C, 67% boys, median age 10 years). There was no significant difference between groups in terms of age, sex, and parental socio-economic levels, nor regarding WISC characteristics.

Compared to NIQ-C, NIQ-N had more school difficulties (77% vs. 29%, *p* < 0.01), higher AESS (median 17 vs. 2, *p* < 0.001) and ISI (median 13 vs. 4, *p* < 0.001) total scores, and higher pathological scores for AESS (96% vs. 14%, *p* < 0.001) and ISI (64% vs. 21%, *p* = 0.01).

NIQ-N had lower sleep efficiency (median 84% vs. 96%, *p* < 0.001), less N2 (median 214 min vs. 254 min, *p* = 0.04), shorter sleep (median 6 min vs. 16 min, *p* < 0.01) and REM (median 4 min vs. 156 min, *p* < 0.001) latencies, and more WASO (median 87 min vs. 22 min, *p* < 0.001) than NIQ-C.

There was no other significant difference between groups. All characteristics are reported in Supplementary Material, Table S2.

### 3.3. Children with HIQ Versus NIQ According to Their Pathological Status

#### 3.3.1. Control Children

In the HIQ-C group, one child complained of agitated sleep, eight children had insomnia (associated with phase shift in one child), one child had phase shift, and two had night terrors (associated with enuresis in one child). In the NIQ-C group, three children complained about insomnia. Overall, HIQ-C had more sleep complaints than NIQ-C (48% vs. 14%, *p* = 0.03).

#### 3.3.2. Children with Narcolepsy

There was no significant difference between HIQ-N and NIQ-N regarding cataplexies (24 vs. 18, *p* = 0.17), sleep paralysis (4 vs. 2, *p* = 0.67), and hallucinations (9 vs. 7, *p* = 1). Regarding sleep comorbidities, 30 children reported parasomnia, 16 complained about insomnia, 6 had experienced somnambulism, and 2 had night terrors. There was no significant difference between HIQ-N and NIQ-N regarding sleep comorbidities (64% vs. 68% had at least one comorbidity, *p* = 0.70).

There was no significant difference between HIQ-N and NIQ-N regarding obesity (48% vs. 59%, *p* = 0.56), BMI (median 21.7 vs. 22.4, *p* = 0.24), BMI z-score (median 1.7 vs. 2.9, *p* = 0.27), OAHI (median 0.5 vs. 0.6, *p* = 0.95), minimal saturation (92.8% vs. 93%, *p* = 1.00), arousal index (median 12 vs. 12, *p* = 0.13), PLMSI (median 0.6 vs. 2.9, *p* = 0.28), PLMSI ≥ 5 (30 vs. 45%, *p* = 0.51). There were 2 children with an OHAI ≥ 5, 1 in each IQ group. HIQ-N had a lower index of desaturation ≥3% than NIQ-N (median 0 vs. 0.05, *p* = 0.04).

There was no significant difference between groups regarding medication at the time of the psychometric evaluation (60% vs. 64%, *p* = 1.00). In the HIQ-N group, 10 children underwent a monotherapy (6 with Modafinil and 4 with Methylphenidate) and 5 underwent a bi-therapy (3 with Methylphenidate and Venlafaxine, 1 with Methylphenidate and Modafinil, and 1 with Modafinil and Venlafaxine). In the NIQ-N groups, 11 children underwent a monotherapy (8 with Modafinil, 2 with Methylphenidate, and 1 with Mazindol) and 3 children underwent a bi-therapy (2 with Methylphenidate and Venlafaxine, and 1 with Methylphenidate and Modafinil).

## 4. Discussion

Regardless of their cognitive profile, children with narcolepsy exhibited sleep and wakefulness characteristics commonly observed in this condition. Both HIQ-N and NIQ-N children had higher sleepiness scores, lower sleep efficiency, shorter sleep and REM latencies, and less stage 2 (in duration and proportion) than HIQ-C and NIQ-C children. In addition, compared to HIQ-C, HIQ-N had less TST and N3 (in duration), more N1 (in proportion), and sleep fragmentation. The two groups of children with narcolepsy were comparable in terms of cataplexies, sleep paralysis, hallucinations, sleep comorbidities, anthropometric characteristics (i.e., obesity, BMI, BMI z-score), PSG parameters (OHAI, minimal saturation, arousal index, and PLMSI) and medication. As previously reported, NIQ-N children had a higher index of desaturation ≥3%, [16].

HIQ-N children did not have the same intellectual profile as HIQ-C children. Their PRI was lower, suggesting a deleterious impact of the pathology, namely of excessive daytime sleepiness and sluggishness, on cognitive functioning. Sleepiness creates an unstable state of vigilance, requiring these children to engage more cognitive resources to maintain an optimal level of vigilance, with an extra-cognitive cost [31] and thus fewer resources available for the other dimensions of the task. This misallocation of cognitive resources due to their constant investment in maintaining adequate attention levels has been explained in functional terms: adolescents with narcolepsy are able to perform as well as healthy controls on a cognitive task, but at the cost of an increased deactivation of the default-mode network [32]. When compensation is no longer possible, sleepiness can result in microsleeps [2], leading to omissions or increased reaction times [33,34], which may be considered as attentional disorders in this population [35,36]. These cognitive specificities could influence the PRI, which involves a combination of complex cognitive processes, with no impact on the PSI, which only includes simple and easily compensated tasks. As has been suggested in adults, individuals with narcolepsy may have cognitive impairments in various subdomains, independently of their IQ [15,33]. However, because HIQ-C perform better, the distinction with HIQ-N might be clearer. This could explain why no differences in the cognitive profile of NIQ children was observed herein, whether they had narcolepsy or not. Another hypothesis is that the developmental characteristics leading to high intellectual functioning and/or functional characteristics are different between HIQ children with and without narcolepsy. Studies in fMRI are needed to explore the differences between children with narcolepsy and controls according to their cognitive profile.

Cognitive abilities seem to interfere with psychosocial adjustment in children with narcolepsy. School difficulties appear to be higher in NIQ-N compared to NIQ-C, which is not the case in children with HIQ. This may be related to the impact of sleepiness on daytime cognitive functioning. The cognitive profile of NIQ-N children could reflect less compensation possibilities than HIQ-N, resulting in a pervasive impact on daily-life [37]. Interestingly, HIQ-N reported fewer conducts disorders than HIQ-C, suggesting that their intellectual abilities might be considered as a “protective factor”—as has been reported regarding the mental health of children with chronic diseases [10]—helping them to better cope with their chronic pathology [38], developing more mature behaviors compared to their peers and thus promoting psychosocial adjustments. Another possibility is that the HIQ-C children who were referred to our center had more psychosocial problems. The HIQ-C group was mostly composed of children who came for school orientation related issues, whereas most NIQ-C children were enrolled in a research study because they did not have sleep disorders. However, this may be representative of what is found in the general population: children with HIQ are at risk of sleep disorders [17]. A recent study found that this risk is increased by 4.67 times in children with higher intellectual potential [39].

The present study has other limitations. The control children underwent their PSG at home, which may not be comparable to the hospital sleep experienced by the children with narcolepsy. Moreover, only a single-night PSG was recorded, which may not reflect usual sleep characteristics. Furthermore, 62% of the children with narcolepsy were on medication at the time of the psychometric assessment, with nevertheless an equal representation of treated subjects in HIQ-N and NIQ-N groups. A recent study explored the evolution of cognitive abilities from diagnosis (without treatment) to one-year follow-up under medication [13] and reported an increase in IQ (especially processing speed and perceptual reasoning) to levels comparable to that of control children. Because treatment decreases symptoms and improves vigilance levels, their performances under treatment can be considered to reflect their full cognitive abilities. Finally, although there was no significant difference between groups concerning parental socio-economic levels, there was a significant prevalence of high socio-economic levels in the overall cohort, which may not be representative of the general population. To conclude, although these results provide a better understanding of the cognitive functioning of children with narcolepsy, it may be specific to this sample. Studies conducted on the intellectual capacities of children with narcolepsy (including this one) are few and carried out on small samples [11,12,14]. Additional studies are needed to validate these initial observations.

## 5. Conclusions

In children with HIQ, those with narcolepsy appear to have a different cognitive profile than controls. A high intellectual potential could act as a protective factor against the impact of the disease on cognitive and adaptive functioning. Conversely, NIQ could predict a greater impact of the condition on children’s daily-life functioning, highlighting the need for a multifactorial management of narcolepsy.

## Data Availability

Data are not available do to ethical and privacy restrictions: consents forms do not allow data utilization by other research teams. Data may be available on request from the corresponding author and after additional and documented consent of parents involved in this study.

## Supplementary Materials

The following supporting information can be downloaded at: www.mdpi.com/article/10.3390/jcm11164681/s1, Table S1: Characteristics of children with HIQ according to their narcolepsy (HIQ-N) or control (HIQ-C) status; Table S2: Characteristics of children with NIQ according to their narcolepsy (NIQ-N) or control (NIQ-C) status; Figure S1: Flowchart of children selection in each group; Brief description of questionnaires. References [26,27,28,29] are cited in Supplementary Materials.
