# Supplementary material for: Sleep and Psychosocial Characteristics of Children with Narcolepsy According to Their Intellectual Profile: A Case–Control Study
Source: J Clin Med. 2022 Aug 10;11(16):4681. doi: 10.3390/jcm11164681 (PMC9410520; doi:10.3390/jcm11164681)
Supplement: Supplementary file 1 [file jcm-11-04681-s001.zip › jcm-1865040-supplementary.pdf]

Supplementary Materials

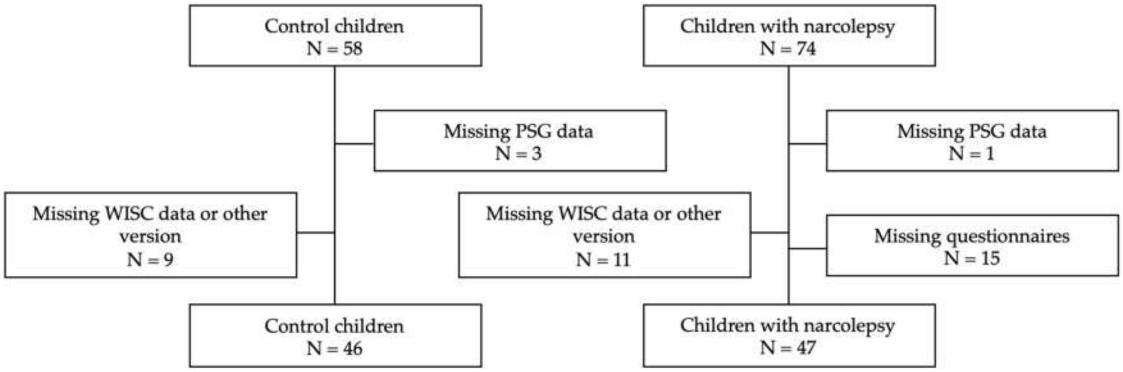

Figure S1. Flowchart describing children selection in each group.

Questionnaires

The Adapted Epworth Sleepiness Scale (AESS) [26]
Evaluation of the daytime sleepiness by an assessment of the risk of falling asleep in 8 daily life situations estimated on a 4-point Likert scale. The total score is the sum of the scores for the 8 items: a higher score represents greater sleepiness and the pathological threshold is higher than 10.

The Insomnia Severity Index (ISI) [27]
Assessment of the insomnia severity in 7 items scored on a 5-point Likert scale ranging from "not at all" to "extremely." The higher the total score, the more severe symptoms. The total score is considered pathological when it is higher than 10.

The Child Depression Inventory (CDI) [28]
Assessment of the depression symptomatology using 27 items scored on a 3-point Likert scale. The abnormal cut-off score is higher or equal to 16.

The Revised Conners Parents Rating Scale (Conners) [29]
Assessment of the behavioral and attention disorders using 48 items. This version is divided in 6 components (i.e., conduct, learning, psychosomatic, impulsivity, anxiety, and hyperactivity), and each component is standardized within age groups. Moderate to severe symptoms were defined with a cut-off above 65, severe symptoms were defined with a cut-off above 75.

Table S1. Characteristics of children with HIQ according to their narcolepsy (HIQ-N) or control (HIQ-C) status

	**HIQ-N** **N=25**	**NA**	**HIQ-C** **N=25**	**NA**	**Statistics**	**95%CI**	***p*-value**
**Demographic characteristics**							
Sex, male, N (%)	10 (40)	0	17 (68)	0	F = 3.11	[0.87; 11.97]	0.09
Age, years	11.5 (6.7–17.6)	0	11.7 (7–15.1)	0	t = 0.69	[−0.90; 1.83]	0.55
Body Mass Index (BMI)	21.7 (15.4–31.6)	0	-	25	-	-	-
BMI z-score	1.7 (−0.5–7.6)	0	-	25	-	-	-
Obesity (BMI z-score > 2), N (%)	12 (48)	0	-	25	-	-	-
Socio-economic level, N (%)		0		5	X^2 = 4.25	-	0.51
Farmers	1 (4)		0 (0)				
Artisans, shopkeepers, CEOs	1 (4)		0 (0)				
Executive and intellectual professions	9 (36)		6 (30)				
Intermediate professions	6 (24)		7 (35)				
Employees	6 (24)		7 (35)				
Workers	0 (0)		0 (0)				
Students and unemployed	2 (8)		0 (0)				
School difficulties, N (%)	5 (20)	0	10 (42)	1	FE = 2.79	[0.69; 12.88]	0.13
**Psychometric characteristics**							
VCI	135 (118–154)	0	143 (124–155)	0	W = 234	[−10.99; 1.99]	0.13
PRI	116 (99–142)	0	124 (96–148)	0	t = −2.04	[−14.39; −0.09]	0.047
WMI	109 (85–130)	2	109 (79–133)	0	t = 0.33	[−6.31; 8.74]	0.75
PSI	109 (76–131)	1	109 (86–143)	1	t = −1.05	[−12.74; 3.99]	0.30
IQ	129 (108–148)	2	131 (115–147)	1	t = −1.67	[−10.33; 0.95]	0.10
SVPD > 15, N (%)	19 (76)	0	16 (64)	0	F = 0.57	[0.13; 2.25]	0.54
SVPD, VCI > PRI	19 (100)	0	16 (100)	0	F = 0	-	1.00
SVPD	22 (5–50)	0	19 (0–44)	0	t = 1.08	[−2.96; 9.76]	0.30
**Sleep characteristics**							
TST, min	463 (324–561)	0	522 (349–623)	0	t = −3.92	[−97.38; −31.34]	<0.001

N1, min	64 (1–137)	0	55 (23–93)	0	t = 1.43	[−4.28; 24.96]	0.16
N1, %	14 (0–28)	0	10 (4–18)	0	t = 2.69	[1.06; 7.46]	0.01
N2, min	178 (24–293)	0	234 (137–333)	0	t = −4.10	[−78.64; −26.76]	<0.001
N2, %	39 (28–65)	0	45 (38–57)	0	t = −2.16	[−7.63; −0.25]	0.04
N3, min	90 (28–142)	0	111 (65–151)	0	t = −2.42	[−32.67; −3.04]	0.02
N3, %	20 (9–33)	0	21 (12–29)	0	t = −0.18	[−3.26; 2.75]	0.86
REM, min	102 (18–190)	0	124 (64–225)	0	t = −1.89	[−40.70; 1.30]	0.07
REM, %	23 (11–39)	0	24 (17–36)	0	t = −0.04	[−3.22; 3.08]	0.97
Efficiency, %	84 (57–91)	0	96 (68–99)	0	W = 55.5	[−15.70; −8.80]	<0.001
Latency, min	6 (0–78)	0	28 (7–103)	0	W = 99.5	[−27; −9]	<0.001
REM latency, min	17 (0–225)	0	131 (56–209)	0	W = 146.5	[−111.50; −45]	<0.001
Arousal index, /h of TST	12 (6–22)	2	10 (5–14)	0	W = 389.5	[0.10; 5.30]	0.04
WASO, min	92 (30–260)	0	23 (7–118)	0	W = 603	[52; 94]	<0.001
OAHI, /h of TST	0.5 (0–19.9)	0	-	25	-	-	-
Minimal oxygen saturation, %	92.8 (69.3–96.3)	1	-	25	-	-	-
Desaturation >3% index, /h	0 (0–1.5)	1	-	25	-	-	-
Periodic limb movement index, /h of TST	0.6 (0–33.9)	5	-	25	-	-	-
Periodic limb movement index > 5 / h, N (%)	6 (30)	5	-	25	-	-	-
**Questionnaires**							
AESS, total	16 (9–23)	0	2 (0–21)	0	W = 602	[12; 16]	<0.001
AESS pathological, N (%)	24 (96)	0	1 (4)	0	F = 0.002	[0; 0.04]	<0.001
ISI, total	12 (4–22)	2	11 (2–18)	2	t = 1.91	[−0.14; 5.80]	0.06
ISI pathological, N (%)	17 (74)	2	12 (52)	2	F = 0.393	[0.09; 1.55]	0.22
CDI, total	9 (0–38)	1	12 (0–36)	1	W = 264.5	[−7; 4}	0.63
CDI pathological, N (%)	5 (21)	1	10 (42)	1	F = 2.66	[0.65; 12.30]	0.21
Conners, total	15 (1–58)	5	33 (1–75)	3	W = 134.5	[−25; −1]	0.03
Conners pathological (>65), N (%)	0 (0)	5	2 (9)	3	F = 0	[0; 5.29]	0.49
Conners pathological (>75), N (%)	0 (0)	5	1 (5)	3	F = 0	[0; 39]	1.00
Conducts disorders, total	43 (39–92)	6	51 (40–99)	3	W = 123	[−14; 0]	0.03
Learning disorders, total	45 (36–98)	6	63 (38–91)	3	W = 141.5	[−21; 0]	0.08

Psychosomatic disorders, total	45 (42–106)	6	62 (42–101)	3	W = 147.5	[−18; 1]	0.11
Impulsivity, total	41 (35–68)	6	53 (35–86)	3	W = 136.5	[−20; 3]	0.06
Anxiety, total	50 (40–78)	6	57 (40–84)	3	W = 149	[−16; 2]	0.12
Hyperactivity, total	48 (35–88)	6	57 (33–92)	3	W = 156.5	[−18; 3]	0.17

CI: confidence intervals; NA: non-available data; F: Fisher Exact Test; t: t-test; X^2: Chi-squared test; W: Wilcoxon test. Values are reported as median (range) and n (%). VCI: Verbal comprehension index; PRI: Perceptual reasoning index; WMI: Working memory index; PSI: Processing speed index; GAI: General abilities index; IQ: Intelligence quotient; SVPD: Significant verbal performance discrepancy; N1: stage 1; N2: stage 2; N3: stage 3; REM: rapid eyes movements; min: minutes; WASO: Wake after sleep inset; OAHI: Obstructive Apnea Hypopnea Index (OAHI); ISI: Insomnia severity scale; CDI: Children depression inventory.

Table S2. Characteristics of children with NIQ according to their narcolepsy (NIQ-N) or control (NIQ-C) status

	NIQ-N N=22	NA	NIQ-C N=21	NA	Statistics	95%CI	*p*-value
**Demographic characteristics**							
Sex, male, N (%)	12 (55)	0	14 (67)	0	F = 1.65	[0.41; 6.90]	0.54
Age, years	12.1 (6.6–14.9)	0	10 (7.3–15.1)	0	W = 278	[−0.60; 2.70]	0.26
BMI	22.4 (15.5–31.6)	0	-	21	-	-	-
BMI z-score	2.9 (−1.8–9)	0	-	21	-	-	-
Obesity (BMI z-score > 2), N (%)							
Socio-economic level, N (%)		0		2	X^2 = 9.33	-	0.14
Farmers	1 (4)		0 (0)				
Artisans, shopkeepers, CEOs	0 (0)		0 (0)				
Executive and intellectual professions	4 (18)		9 (47)				
Intermediate professions	5 (23)		3 (16)				
Employees	6 (27)		7 (37)				
Workers	3 (14)		0 (0)				
Students and unemployed	3 (14)		0 (0)				
School difficulties, N (%)	17 (77)	0	6 (29)	0	F = 0.12	[0.02; 0.55]	0.002
**Psychometric characteristics**							
VCI	113 (86–128)	0	114 (82–128)	0	W = 207.5	[−10; 4]	0.58
PRI	107 (75–128)	0	104 (77–124)	0	t = −0.35	[−9.47; 6.70]	0.73
WMI	99 (73–115)	0	96 (60–115)	1	W = 208	[−12; 9]	0.77
PSI	96 (64–124)	0	100 (64–131)	1	t = −1.44	[−18.74; 3.16]	0.16
IQ	103 (73–122)	0	108 (72–127)	1	t = −0.84	[−12.37; 5.08]	0.40
SVPD > 15, N (%)	6 (27)	0	6 (29)	0	F = 1.07	[0.23; 4.99]	1.00
SVPD, VCI > PRI	5 (83)	0	5 (83)	0	F = 0.94	[0.18; 4.96]	1.00
SVPD	11 (1–26)	0	10 (1–30)	0	t = 0.59	[−3.09; 5.68]	0.55
**Sleep characteristics**							
TST, min	485 (269–616)	0	526 (348–654)	0	t = - 1.80	[−85.92; 4.85]	0.08
N1, min	66 (23–136)	0	58 (27–149)	0	W = 266	[−9; 21]	0.40
N1, %	14 (7–27)	0	11 (5–27)	0	W = 290.5	[−0.80; 4.90]	0.15
N2, min	214 (94–291)	0	254 (140–299)	0	t = −2.08	[−62.84; −0.96]	0.04
N2, %	43 (28–56)	0	46 (33–58)	0	t = −1.34	[−6.80; 1.39]	0.19
N3, min	98 (46–198)	0	113 (73–149)	0	t = −1.89	[−31.80; 1.08]	0.07
N3, %	20 (10–44)	0	21 (13–35)	0	W = 194	[−5.60; 2]	0.38

REM, min	102 (29–176)	0	100 (62–178)	0	t = 0.26	[−17.48; 22.69]	0.79
REM, %	20 (11–31)	0	18 (12–32)	0	t = 1.17	[−1.33; 4.98]	0.25
Efficiency, %	84 (53–95)	1	96 (83–98)	0	W = 35.5	[−14; −6]	<0.001
Latency, min	6 (0–77)	0	16 (7–61)	0	W = 109	[−17; −5]	0.003
REM latency, min	4 (0–211)	0	156 (50–243)	0	W = 57	[−153.5; −71]	<0.001
Arousal index, /h of TST	12 (5–19)	2	9 (5–15)	0	t = 1.48	[−0.59; 3.77]	0.15
WASO, min	87 (26–216)	0	22 (9–88)	0	W = 426	[36; 79]	<0.001
OAHI, /h of TST	0.6 (0–9.4)	0	-	21	-	-	-
Minimal oxygen saturation, %	93 (50.4–96)	0	-	21	-	-	-
Desaturation >3% index, /h	0.1 (0–19.3)	0	-	21	-	-	-
Periodic limb movement index, /h of TST	2.9 (0–25.6)	2	-	21	-	-	-
Periodic limb movement index > 5 / h, N (%)	9 (45)	2	-	21	-	-	-
**Questionnaires**							
AESS, total	17 (9–23)	0	2 (0–16)	0	W = 452	[11; 16]	<0.001
AESS pathological, N (%)	21 (96)	0	3 (14)	0	F = 0.01	[0; 0.1]	<0.001
ISI, total	13 (2–20)	0	4 (1–17)	2	W = 341.5	[3; 10]	0.001
ISI pathological, N (%)	14 (64)	0	4 (21)	2	F = 0.16	[0.03; 0.73]	0.01
CDI, total	10 (5–30)	1	10 (1–27)	2	W = 222.5	[−3; 5]	0.54
CDI pathological, N (%)	4 (19)	1	6 (32)	2	F = 1.93	[0.37; 11.37]	0.47
Conners, total	19 (1–41)	0	13 (0–75)	0	W = 267	[−4; 12]	0.39
Conners pathological (>65), N (%)	0 (0)	0	2 (10)	0	F = 0	[0; 5.04]	0.23
Conners pathological (>75), N (%)	0 (0)	0	1 (5)	0	F = 0	[0; 35.46]	0.48
Conducts disorders, total	51 (39–78)	0	46 (19–99)	0	W = 299.5	[−1; 11]	0.10
Learning disorders, total	57 (38–78)	0	47 (12–95)	0	W = 293.5	[−3; 18]	0.13
Psychosomatic disorders, total	53 (42–82)	0	45 (2–94)	0	W = 266.5	[−2; 10]	0.39
Impulsivity, total	47 (35–72)	0	45 (10–76)	0	W = 247.5	[−6; 8}	0.70
Anxiety, total	50 (40–68)	0	49 (8–89)	0	W = 214.5	[−12; 6]	0.70
Hyperactivity, total	50 (33–76)	0	43 (24–90)	0	W = 290	[−2; 14]	0.16

CI: confidence intervals; NA: non-available data; F: Fisher Exact Test; t: t-test; X^2: Chi-squared test; W: Wilcoxon test. Values are reported as median (range) and n (%). BMI: Body Mass Index; VCI: Verbal comprehension index; PRI: Perceptual reasoning index; WMI: Working memory index; PSI: Processing speed index; GAI: General abilities index; IQ: Intelligence quotient; SVPD: Significant verbal performance discrepancy; N1: stage 1; N2: stage 2; N3: stage 3; REM: rapid eyes movements; min: minutes; OAHI: Obstructive Apnea Hypopnea Index (OAHI); ISI: Insomnia severity scale; CDI: Children depression inventory.
